# Improving depression-like behaviors caused by diabetes is likely to offer a new perspective for the treatment of non-healing chronic wounds

**DOI:** 10.3389/fnbeh.2024.1348898

**Published:** 2024-02-19

**Authors:** Zhiqin Dong, Jijin Wu, Hanchen Cao, Jinqiang Lu

**Affiliations:** ^1^Department of Plastic Surgery, The First Affiliated Hospital of Jinan University, Guangzhou, Guangdong Province, China; ^2^Key Laboratory of Regenerative Medicine, Ministry of Education, Guangzhou, Guangdong Province, China; ^3^Physiology Department, School of Medicine, Jinan University, Guangzhou, Guangdong Province, China; ^4^Department of Plastic Surgery, The Fifth Affiliated Hospital of Jinan University, Guangzhou, Guangdong Province, China

**Keywords:** chronic wound healing, depression, diabetes, brain, the lateral habenula

## Abstract

**Background:**

Three phases are often involved in the intricate process of wound healing: inflammatory exudation, cell proliferation, and tissue remodeling. It is challenging for wounds to heal if conditions like ischemia, persistent pressure, infection, repetitive trauma, or systemic or localized illnesses arise during the healing process. Chronic wounds are persistent injuries that do not follow the normal healing process and fail to progress through the stages of healing within a reasonable timeframe, like diabetic ulcers, vascular ulcers, pressure sores, and infectious wounds. Various factors affect chronic wound healing. A large body of research has illuminated that psychological distress may often be related to wound healing in clinical settings. Our observations have indicated that the pace of wound healing in diabetic mice is generally slower than that of healthy mice, and mice induced by streptozotocin (STZ) and fed a high-fat diet generally exhibit depression-like behavior. Our experiment delves into whether there is an inherent correlation and provides new ideas for clinical treatment to promote wound healing.

**Methods:**

In order to explore the relationship between diabetes, depression, and wound healing, we observed wound healing through HE staining, Masson's trichrome staining, and IHC staining for CD31 and detected the depressive condition through behavioral tests. Then, RT-PCR was used to detect the mRNA expression levels of α-SMA, Col1, CD31, and VEGF in wound tissue. Finally, the related brain areas were regulated through chemical genetic methods and the process of wound healing was observed.

**Conclusion:**

It has been observed that the lateral habenula (LHb) areas are associated with depression-like behavior induced by diabetes. Inhibiting LHb neuronal activity mitigates these depressive symptoms and enhances wound healing. Refractory wounds can be improved by considering patients' emotional issues from a broad standpoint, which provides fresh concepts for potential clinical treatments in the future.

## 1 Introduction

The healing of chronic wounds remains a major challenge, with slow healing rates and high recurrence rates posing a substantial and pervasive global encumbrance (Morbach et al., 2012). Traditional treatment methods including local wound care, drug therapy, and even surgical intervention, have been shown to be of limited effectiveness. These approaches merely offer symptomatic relief at a macroscopic level, falling short of actively orchestrating wound healing by regulating the underlying biological process. A large body of research has illuminated that psychological distress may often be related to wound healing in clinical settings (Gurtner et al., 2008). As living standards continue to improve, more and more people are suffering from so-called “diseases of affluence,” such as diabetes and depression. The interplay between these two afflictions is intricate and diverse, with evidenced studies showing that people with type 1 and type 2 diabetes are at higher risk of depression than people without diabetes (Campayo et al., 2011; Dixon and Edmonds, 2021).

In our study, we have discerned that the majority of STZ-induced diabetic mice developed depression after 1 month of high-fat diet feeding. Depression is believed to arise from maladaptive alterations in specific brain circuits (Zahm and Root, 2017), and in recent years, the lateral habenula (LHb) has emerged as a key brain region related to depression (Hikosaka, 2010; Zahm and Root, 2017). Emerging evidence from studies involving rodents, non-human primates, and humans suggests that abnormal activity in the LHb is linked to depressive symptoms, including feelings of helplessness, anhedonia, and heightened negative attention (Wu et al., 2022). A lot of studies have reported that LHb is susceptible to being activated by various stressors and negative emotional stimuli (Li et al., 2011), indicating its key role in orchestrating stress and aversive behavioral responses under normal physiological conditions. Recordings in macaque monkeys have revealed that LHb neurons encode negative reward prediction error (RPE), exhibiting increased firing when animals failed to receive an expected reward (“disappointed”) or when they received a cue predicting aversive stimuli (Wirtshafter et al., 1994; Ng et al., 2015).

Our experimental hypothesis posits that amelioration of depression induced by diabetes can be achieved through the regulation of the LHb brain region, consequently accelerating the process of wound healing. In order to scrutinize the validity of this proposition, meticulously designed *in vivo* experiments were undertaken.

## 2 Materials and methods

### 2.1 Experimental animals

Clean-grade male C57/BL6 mice provided by the Guangdong Medical Laboratory Animal Center, at nearly 8 weeks old and weighing approximately 24 g, were provided by the Animal Center of Jinan University. All animals were maintained under controlled conditions, with a room temperature of 22–25°C and a 12-h alternating light-dark cycle. Three mice were kept in each cage after the wound model was prepared. The entire experimental process adhered to the ethical standards of experimental animals, and the laboratory personnel had the approval of the Laboratory Animals Ethics Committee of Jinan University.

### 2.2 Intracranial injection and diabetic wound models

We used chemogenetic methods to inhibit the expression of LHb neurons in a diabetic mouse model. Long-term inhibition of LHb neurons significantly decreased depression-like behaviors. To infect LHb neurons with eYFP and hM4Di-eYFP, AAV2/9-hSyn-eYFP (Taitool Bioscience) and AAV2/9-hSyn-hM4Di-eYFP (Taitool Bioscience) were injected into the LHb [0.1 lL/injection; anteroposterior (AP):−1.6 mm; mediolateral (ML): ±0.3 mm; dorsoventral (DV):−3.05 mm].

After 21 days of the expression of the virus, the mice were intraperitoneally injected with streptozotocin for 2 consecutive days to induce the T2DM model, and the mice were fed a high-fat diet for a month. The average weight of T2DM mice was 20.7 g, and the weight of healthy mice was 28.8 g. The average blood glucose of the T2DM mice finally selected in the experiment was 29.3 mmol/L, whereas the normal mice exhibited an average blood glucose level of 8.7 mmol/L (Supplementary Figure 1). Subsequently, a full-thickness cutaneous wound of 1 cm diameter was created on the dorsal surface. Wound size was measured from digital photographs 0, 3, 7, and 14 days after surgery and quantified using ImageJ software.

### 2.3 Injection site verification

First, 0.9% normal saline was perfused through the myocardium, then 4% paraformaldehyde (PFA) and 0.1 mol/L phosphate buffered saline (PBS) were added. Brains were excised and further fixed with 4% PFA overnight at 4°C. It was then soaked in a solution containing 30% sucrose for cryoprotection and subsequently cut into slices using a cryostat (CM1900, Leica Microsystems, Bannockburn, USA). To confirm the injection sites of viruses encoding fluorescent proteins, fluorescence microscopy was used to examine coronal brain sections (Axioimager Z2 microscope, Zeiss, Oberkochen, Germany). Only the mice with verified injection sites were utilized for analysis.

### 2.4 Physiological recordings from brain slices

To prepare the brain slices, the mice were given isoflurane anesthesia. Coronal sections (250 μm thick) containing LHb were sectioned in frozen artificial cerebrospinal fluid (ACSF: 119 mmol/L NaCl, 2.5 mmol/L KCl, 1 mmol/L NaH2PO4, 11 mmol/L glucose, 26.2 mmol/L NaHCO3, 2.5 mmol/L CaCl2, 1.3 mmol/L MgCl2, 290 mOsm, at pH 7.4). The brain slices were allowed to recover for 1 h at room temperature in N-methyl-D-glucamine-based ACSF (93 mmol/L N-methyl-D- glucamine (NMDG), 93 mmol/L HCl, 2.5 mmol/L KCl, 1.2 mmol/L NaH2PO4, 30 mmol/L NaHCO3, 25 mmol/L D-glucose, 20 mmol/L HEPES, 5 mmol/L Na-ascorbate, 2 mmol/L thiourea, 3 mmol/L Na-pyruvate, 10 mmol/L MgSO4, and 0.5 mmol/L CaCl2, pH 7.35 with NMDG or HCl). After the recovery period, the slices were introduced into the recording chamber and subjected to a continuous flow of ACSF.

To measure the function of chemogenetic viruses, neurons expressing hM4Di-eYFP in LHb were recorded. For chemogenetic inhibition, eYFP-labeled neurons were injected with a 50 pA current, and the number of activated action potentials was calculated as the baseline. Then, 10 μM CNO (C0832, Sigma) was added to ACSF for 10 min and the action potentials activated by 50 pA current injection were recorded. Finally, the CNO was washed out, and the activated action potentials were recorded. All recordings were performed using a Multiclamp 700B amplifier (Molecular Devices). Traces were low-pass filtered at 2 kHz and digitized at 10 kHz. The pipette resistance ranged from 4 to 6 M. Stable whole-cell recordings were obtained when the access was maintained below 25 MΩ, and basic electrophysiological properties were recorded. Offline data analysis was performed using Clampft10.0 software (Molecular Devices).

### 2.5 Immunohistochemistry

The animals underwent anesthesia before receiving an intracardial infusion of 0.9% saline, followed by 4% PFA in PBS. The brains were carefully removed thereafter. For c-Fos labeling, the same methods used for GABA (Sigma) labeling were utilized, with the exception that the main antibody was swapped with an antibody specific to c-Fos (Cell Signaling Technology, Boston, USA), and the secondary antibody was replaced with goat anti-rabbit Alexa Fluor 488 (Jackson ImmunoResearch, West Grove, USA). Finally, all slices were washed in 0.1 mol/L PBS and cover-slip mounted in an antifade aqueous mounting medium containing DAPI (Roche, Basel, Switzerland).

For anti-CD31 labeling, sections were incubated overnight at 4°C and then treated with the secondary antibody, horseradish peroxidase (HRP)-labeled goat anti-rabbit IgG (1:1000, Wuhan Boster Biological Technology, Wuhan, China), for 30 min at room temperature. Each segment was then immersed in a diaminobenzidine solution (ZSGB-Bio, China) and the nuclei were counterstained with hematoxylin. Subsequently, the sections were photographed using a microscope.

### 2.6 Behavioral paradigms

#### 2.6.1 Sucrose preference test

Mice were tested for preference for a 2% sucrose solution (Sucrose, Sigma-Aldrich) using a two-bottle choice procedure. Each animal was provided with two bottles, one containing the source and the other tap water. The quantities of source and water consumed were recorded every 24 h. To prevent potential location preference for drinking, the positions of the bottles were alternated every 24 h. Food and water were available *ad libitum* prior to the SPT. The preference for the sucrose solution was determined as the percentage of sucrose solution ingested relative to the total intake.

#### 2.6.2 Forced swimming test

Mice were immersed in a water cylinder for about 6 min. Animal behavior was monitored using video tracking from a lateral perspective. The time each animal spent immobile during the test was counted online by two independent observers in a blinded manner. Immobility was defined as no active movement except that needed to keep the animal from drowning.

#### 2.6.3 Tail suspension test

Mice were suspended via adhesive tape attached to their tails for 6 min. Animal behavior was captured on video from a lateral view. The amount of time spent immobile was counted online by two independent observers in a blinded manner. Immobility was defined as the absence of all active motion.

#### 2.6.4 Open field test

Motor activity was evaluated via an open-field test in a plastic box. Mice were placed in the center of the box and given 15 min to explore the arena in dim light. An infrared camera positioned above the cage captured all movement throughout the final 10 min, and movement was measured using Ethovision XT software (Noldus, Wageningen, Netherlands).

### 2.7 Histological assessment

Tissue samples from the wound (including 2 mm of the surrounding skin) were harvested on day 14. The sections of the wound tissue were stained with hematoxylin and eosin (H&E) and with Masson's trichrome following the manufacturer's protocol (Wuhan servicebio technology Co., Ltd., Wuhan, China) to detect the reepithelization tissue formation and collagen deposition, respectively. The length of the epithelial gap was determined according to the criteria described previously.

### 2.8 RNA isolation and quantitative real-time PCR

Total RNA was extracted using a total RNA extraction kit (cat. No. R1200, Solarbio, Beijing, China) and reverse transcribed into cDNA using a PrimeScript RT reagent kit (cat. No. RR014B, Takara Bio, Kusatsu, Japan). qRT-PCR was carried out using SYBR Green Master Mix (cat. No. 11201ES03, Yeasen, Shanghai, China) on a CFX384 real-time PCR system (Bio-Rad Laboratories, Hercules, CA, USA). Relative mRNA expression was quantified using the 2–ΔΔCt method.

### 2.9 Statistical analysis

Mice were randomly grouped in all experiments, GraphPad Prism software 8 and Image J were used to analyze the data. The normality of the data was assessed using the Shapiro–Wilk test. Data that adhered to a normal distribution were expressed as mean ± standard deviation (SD). Student's test (two groups) or analysis of variance (ANOVA, three or more groups) was utilized to determine significant differences among groups when the data were normally distributed. Statistical significance was set at ^*^P < 0.05, ^**^P < 0.01, and ^***^P < 0.001.

## 3 Result

### 3.1 Wounds in diabetic mice healed more slowly

**Figure 1:**
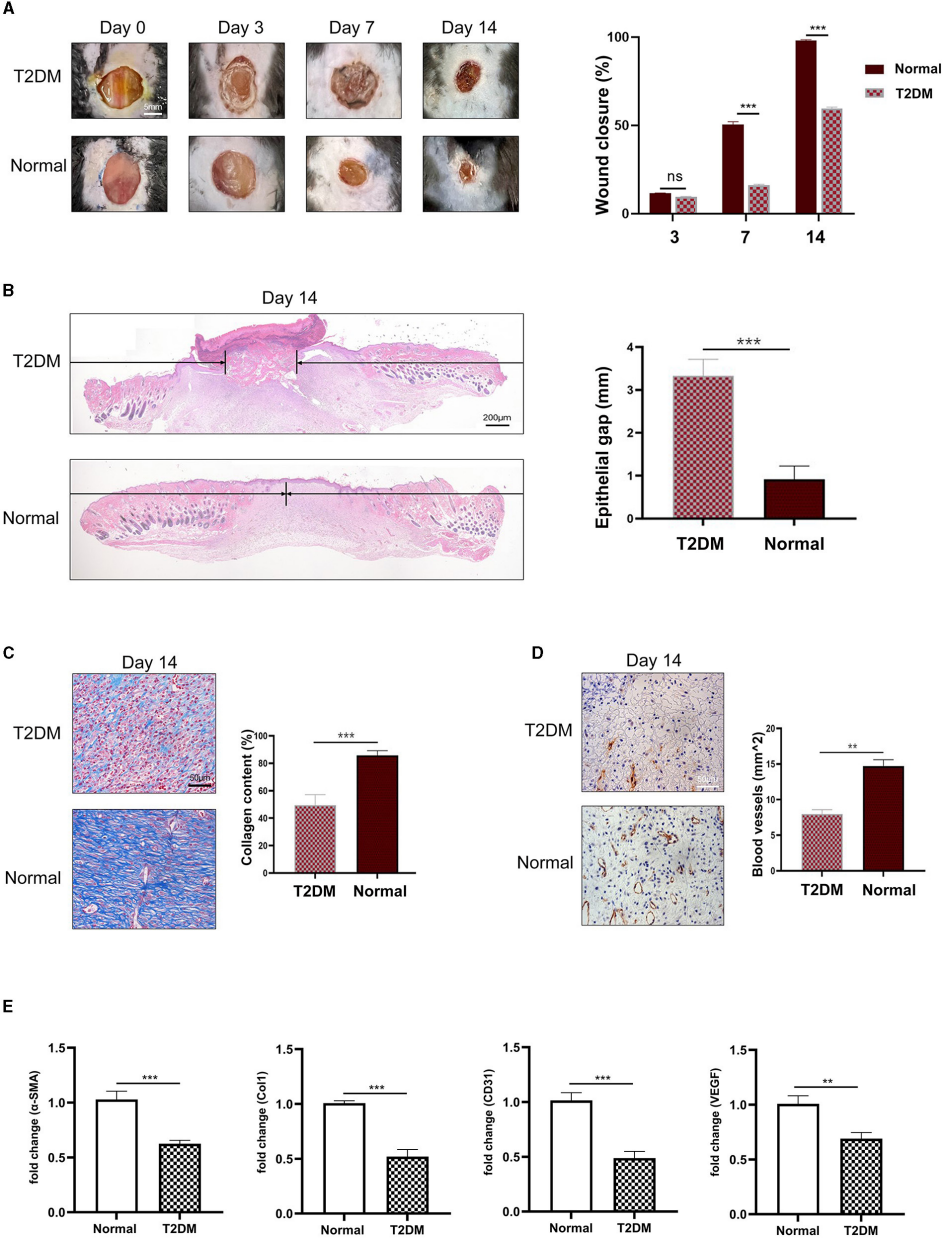
Mice with diabetes had slower wound healing. **(A)** Representative images of wound after operation and wound closure rate in different groups (*n* = 3). **(B)** HandE-stained sections of full-thickness skin wound on day 14, and the length of epithelial gaps (*n* = 3). **(C)** Collagen deposition was assessed by Masson's trichrome on day 14 (*n* = 3). **(D)** IHC staining of CD31 showed more blood vessels in healthy mice (*n* = 3). **(E)** The mRNA expression of α-SMA, Col1, CD31, and VEGF in wound tissue (*n* = 3). **P < 0.01, ***P < 0.001.

A total of 18 mice were randomly divided into two groups, one was the T2DM experimental group and the other was the control group. After the T2DM model was established, the full-thickness skin wound was made on the back. The area of the cutaneous wound was monitored every day, we found that compared with healthy mice, the wound in diabetic mice healed more slowly (Figures 1A, B). Through Masson's trichrome staining, less collagen deposition was observed in the wound in diabetic mice (Figure 1C). IHC staining for CD31 (Figure 1D) showed an increased number of newly formed and mature blood vessels in the dermal defect. Wounds in diabetic mice expressed less angiogenesis compared to the healthy mice. Finally, we employed qRT-PCR analysis to detect the mRNA expression of related genes in the wound tissue. The results showed that compared with healthy mice, the relative expression levels of α-SMA, Col1, CD31, and VEGF mRNA in the wound tissue of diabetic mice were significantly reduced (Figure 1E).

### 3.2 Diabetes-induced depression-like behaviors in mice related to LHb brain region

**Figure 2:**
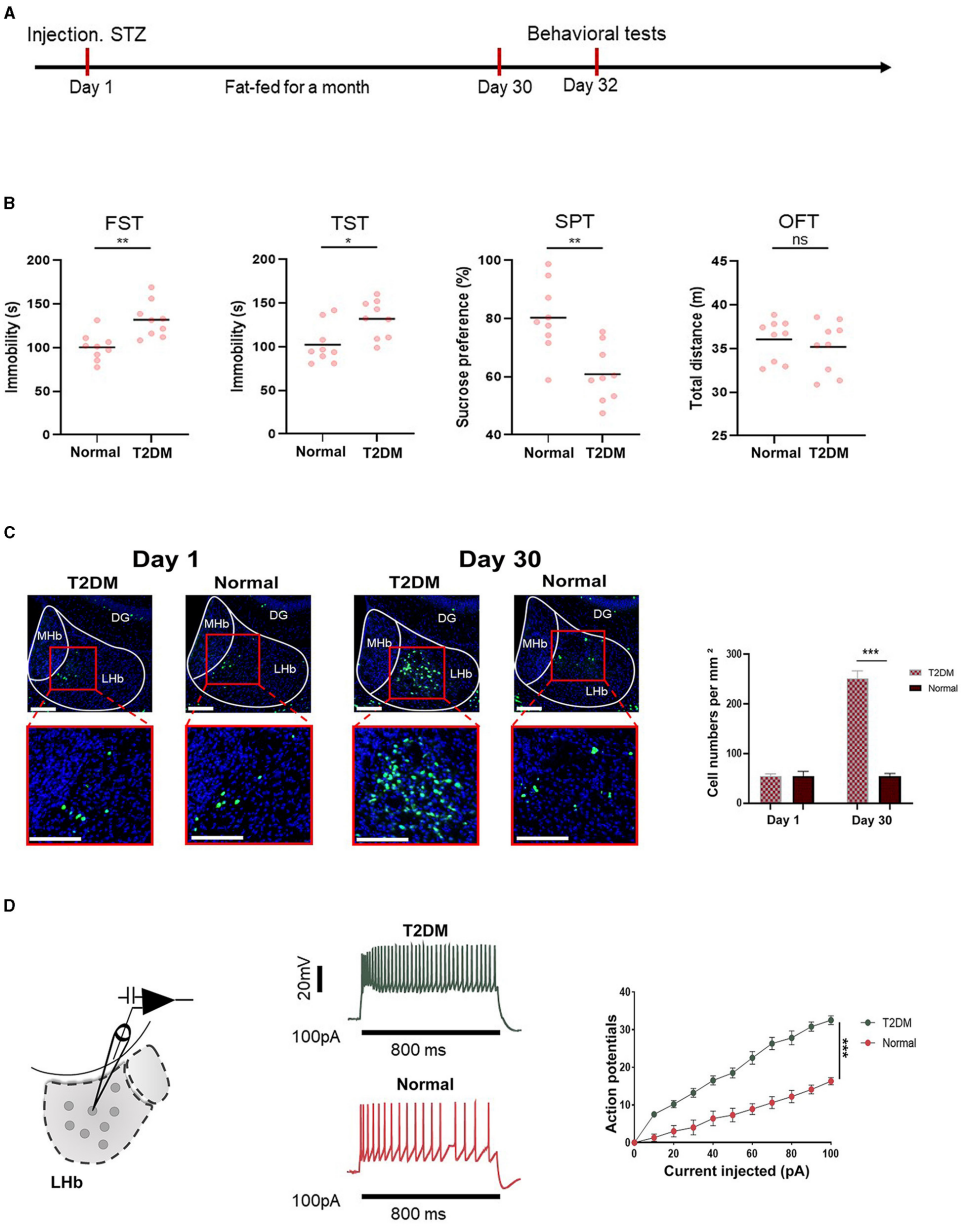
Depression-like behaviors were related to the LHb region. **(A)** Timeline of the experiment. **(B)** The quantification of depression-like behaviors of T2DM and healthy mice (*n* = 9). **(C)** Representative images of the c-Fos expression in the LHb were obtained from T2DM and healthy groups on day 1 and day 30 (*n* = 5). **(D)** Current-evoked action potentials in LHb neurons of mice from the T2DM group (*n* = 10 cells from 5 mice) and healthy group (*n* = 10 cells from 5 mice). *P < 0.05, **P < 0.01, ***P < 0.001.

Figure 2A shows the timeline of behavioral tests, which were designed to determine whether the mice had depression. Figure 2B shows that diabetes significantly increased depression-like behaviors tested in the sucrose preference test (SPT), forced swimming test (FST), tail suspension test (TST), and open field test (OFT). To investigate whether depression in diabetic mice was related to the LHb brain region, we analyzed the expression of c-Fos, a proxy for neuronal activity, on days 1 and 30 in diabetic and healthy mice. Remarkably, we observed that the number of c-Fos+ LHb cells increased significantly in diabetic mice on day 30. In contrast, no significant differences were observed in healthy mice between day 30 and day 1 (Figure 2C). To explore the influence of diabetes on LHb neuronal activity, we performed the whole cell patch clamp recording on LHb neurons after STZ injection and high-fat diet feeding for a month. We found that diabetic mice had increased neuronal firing of LHb neurons compared to healthy mice (Figure 2D).

### 3.3 Inhibition of LHb alleviated depression-like behaviors induced by diabetes

**Figure 3:**
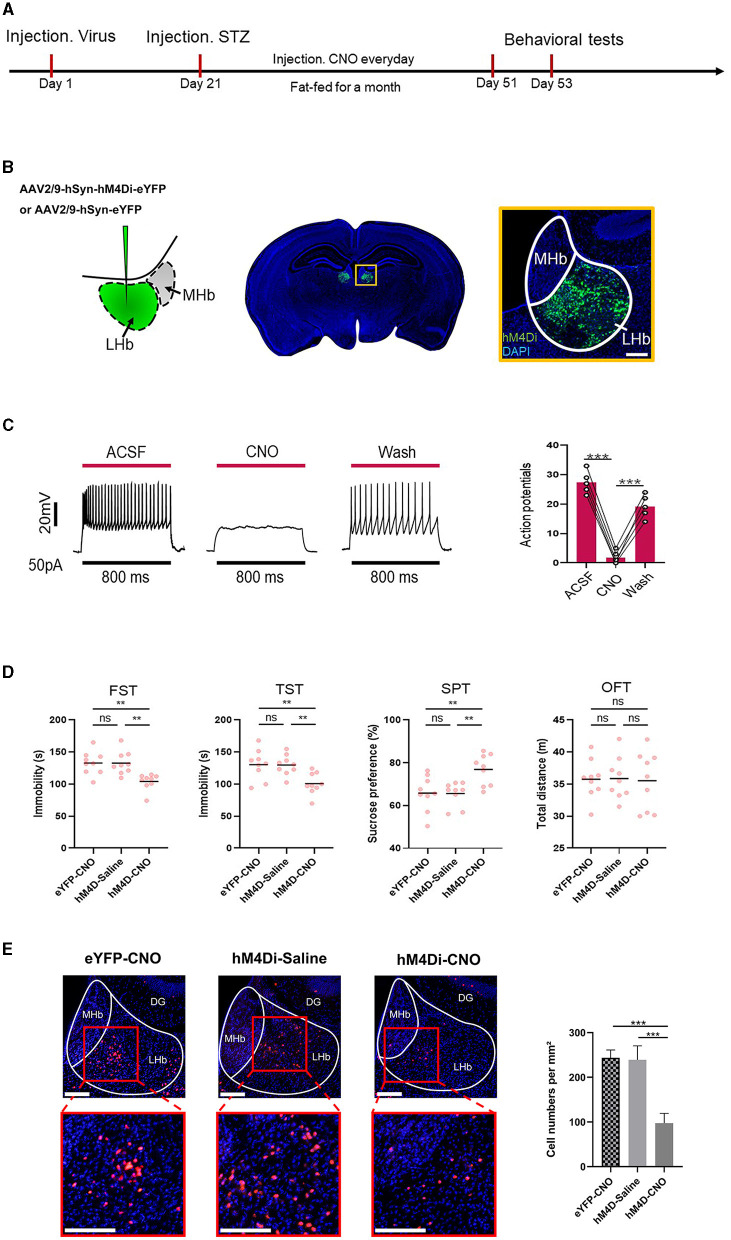
Inhibiting LHb neurons alleviated depression-like behaviors induced by diabetes. **(A)** Timeline of the experiment. **(B)** Scheme for specific infection of LHb neurons with hM4Di or eYFP. **(C)** Current-evoked action potentials in a representative hM4Di-infected LHb neurons recorded before, during, and after CNO perfusion (10 μM). **(D)** Depression-like behaviors in different experimental groups (*n* = 9). The changes in c-Fos+ cells following LHb neuron inhibition. **P < 0.01, ***P < 0.001. **(E)** After inhibition of LHb neurons, the expression of the the percentage of c-Fos+LHb cells.

The timeline of the experiment is shown in Figure 3A. To further determine whether LHb was the critical substrate for the alleviation of depression-like behaviors induced by diabetes, we used chemical genetic approaches to reduce the activity of LHb neurons. On the first day, the virus AAV2/9-hSyn-eYFP and AAV2/9 hSyn-hM4Di-eYFP were injected into the LHb. After 21 days of the expression of the virus, the mice were intraperitoneally injected with streptozotocin to induce the T2DM model. Then, the mice were fed a high-fat diet for a month and CNO (1 mg/kg) was administered via intraperitoneal injection (i.p.) for 30 consecutive days (Fu et al., 2023). We then used some behavior tests, as before, to explore the depression-like behaviors of mice. Figure 3B shows the specific site of virus injection. Physiological recordings showed that CNO inhibited neuronal activity (Figure 3C). Finally, through the behavioral tests, we found that the inhibition of LHb alleviated depression-like behaviors induced by diabetes (Figure 3D). Figure 3E shows changes in c-Fos+ cells following LHb neuron inhibition. The outcome showed that when LHb neurons were suppressed, the expression of c-Fos+ cells sharply decreased.

### 3.4 Inhibition of LHb neurons in diabetic mice accelerated wound healing

**Figure 4:**
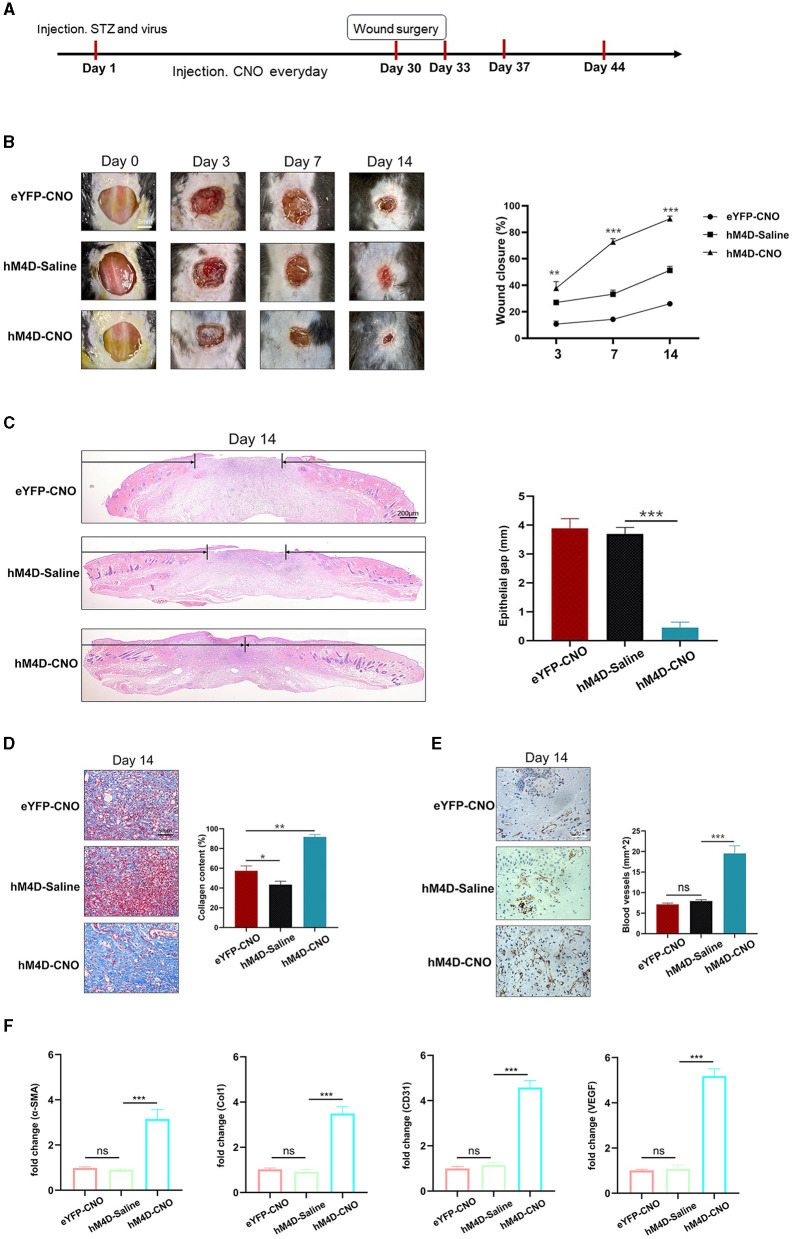
Inhibiting LHb neurons accelerated the process of wound healing. **(A)** Timeline of the experiment. **(B)** Representative images of wound areas in different groups (*n* = 3). **(C)** H&E-stained sections of full-thickness skin wound on day 14, and the length of epithelial gaps (*n* = 3). **(D)** Collagen deposition was assessed by Masson's trichrome on day 14 (*n* = 3). **(E)** IHC staining of CD31 showed more blood vessels in the hM4D-CNO group (*n* = 3). **(F)** The expression of VEGF, Col1, CD31, and α-SMA mRNA in wound tissue by qRT-PCR analysis. *P < 0.05, **P < 0.01, ***P < 0.001.

Figure 4A shows the timeline of the experiment. After the establishment of the diabetic model and creation of full-thickness wounds, daily monitoring indicated that inhibiting LHb neurons in diabetic mice expedited wound healing (Figures 4B, C).

The mice treated with hM4D-CNO were observed to have tremendous collagen deposition and large wavy collagen fibers in the dermis, as determined by Masson's trichrome staining (Figure 4D). CD31 showed that the mice treated with hM4D-CNO had more angiogenic formation compared to the other groups (Figure 4E). Then, we used qRT-PCR analysis to find the associated genes' mRNA expression in the wound tissue. As per Figure 4F, the wound tissue of the hM4D-CNO group exhibited significantly higher levels of relative expression of α-SMA, Col1, CD31, and VEGF mRNA compared to the other groups. It was indicated that inhibiting LHb neurons in diabetic mice would accelerate the process of wound healing.

## 4 Discussion

Type 2 diabetes mellitus (T2DM), depression, and chronic wounds are significant public health and socioeconomic issues (Wilson et al., 2023). Some researchers have reported that one in four individuals with T2DM is affected by clinically significant depression (Semenkovich et al., 2015). Conversely, diagnosis of T2DM increases the risk of incident depression. This linkage reflects a shared etiology consisting of complex bidirectional interactions among multiple variables, a process that may include weight gain, inflammation, autonomic and neurohormonal dysregulation, and structural changes to the hippocampus (Brieler et al., 2016). It is well known that the high glucose microenvironment caused by diabetes can impair normal cellular function, suppress the immune system, and lead to neurological and vascular damage (Wilkinson and Hardman, 2020). All of these factors combined make diabetes individuals more vulnerable to problems with wound healing, which makes the process of healing wounds more complex and difficult. Rarely have studies explored the interconnection of T2DM, depression, and chronic wounds. Since there is an indescribable and inseparable relationship between the three, we speculate that depression is likely to be closely related to wound healing. A substantial body of research has reported that psychological distress may be related to wound healing in clinical settings (Martin and Nunan, 2015; Yu et al., 2019). While the detrimental systemic effects of psychological stress are well established, it is only in recent decades that we have started to explore the biochemical, microbial, and physiological impacts of stress diseases on chronic wounds. Within this extensive range, the subject of depression has attracted particular attention (Mirrione et al., 2014; Yang et al., 2018; Ndalman et al., 2019).

Among the various neural circuits implicated, the lateral habenula (LHb) has recently been recognized as a convergent hub (Matsumoto and Hikosaka, 2007). It integrates information related to value, sensory input, and experience dependence to mediate diverse motivational processes and contribute to the pathophysiology of depression (Roy and Lloyd, 2012; Fakhoury, 2017; Nuno-Perez et al., 2018). This study has revealed that the modulation of the LHb brain region can improve depression-like behavior in mice, consequently enhancing the process of wound healing. This study can be summarized as follows: (1) the differences in wound healing between diabetic mice and healthy mice were observed; (2) the activity of neurons in the LHb brain region of diabetic mice and healthy mice was monitored; (3) after inhibiting the activity of LHb neurons in diabetic mice, whether this intervention exerted an influence on the process of wound healing was observed.

Initially, we found that the wounds of diabetic mice healed more slowly. We conducted behavioral tests on the mice and found that depression-like behaviors were common in STZ-induced diabetic mice. Moreover, we discerned the brain slices of diabetic mice and found that compared with healthy mice, the number of c-fos LHb neurons in diabetic mice was significantly increased. Through the whole cell patch clamp recording on LHb neurons, we found that STZ-induced diabetic mice had increased neuronal firing of LHb neurons compared to healthy mice.

We then inhibited the activity of LHb neurons by continuously injecting CNO every day and conducted behavioral verification. Notably, we observed a tangible enhancement in the depression-like behaviors of the mice post-inhibition of LHb neuronal activity. In the final phase of our investigation, a full-thickness wound with a diameter of 1 cm was dug on the back of the mouse *for vivo* verification. Compared with the control group, HE and Masson staining observed accelerated wound healing and increased collagen deposition in the CNO group. CD31 showed that the inhibition of LHb neurons likely increased new blood vessels, and PCR results showed that the mRNA levels of α-SMA, Col1, CD31, and VEGF were significantly increased in CNO-treated mice. In conclusion, we noted that the wound healing speed was accelerated after the inhibition of the activity of LHb neurons. This is consistent with our previous hypothesis.

**Figure 5:**
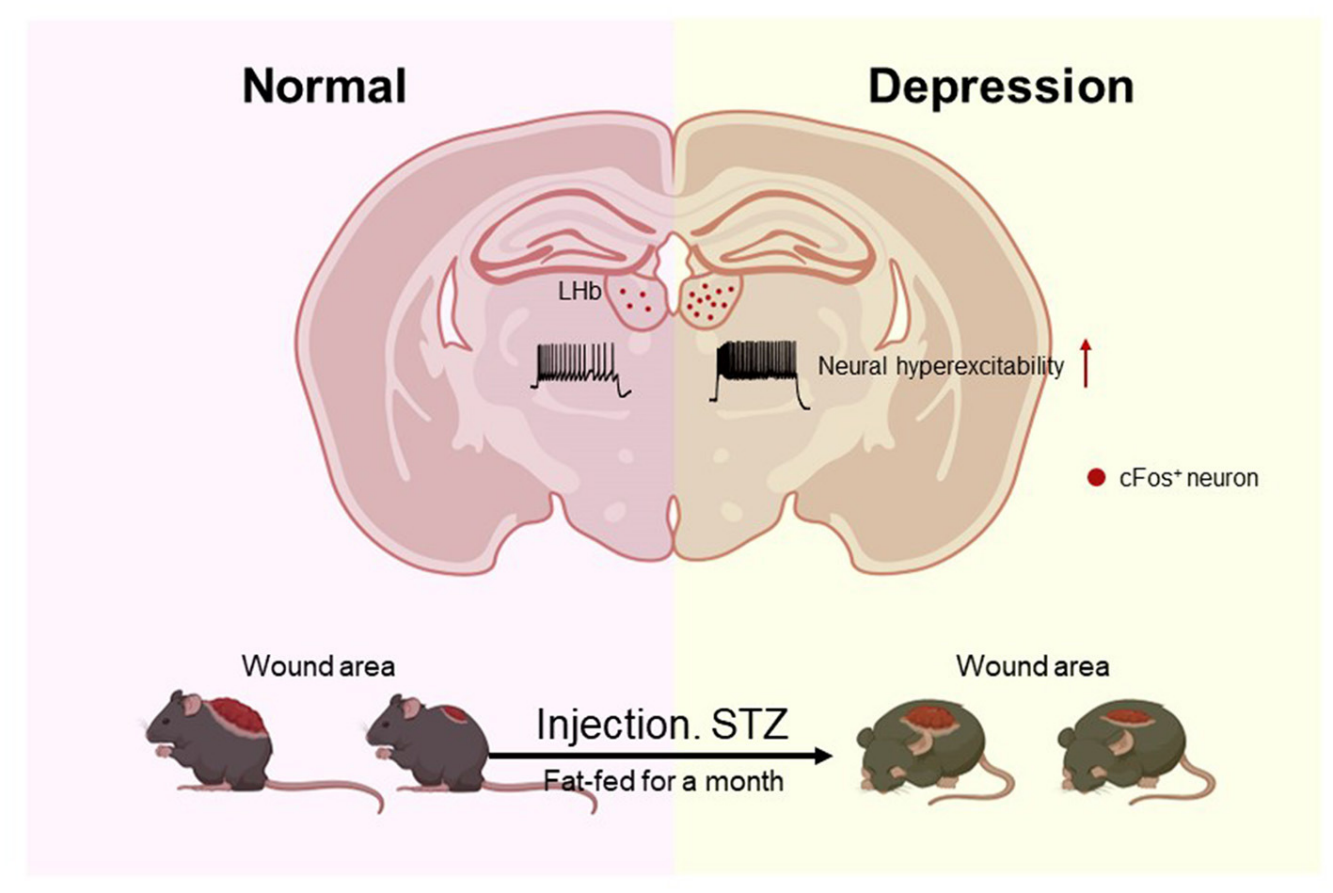
Mice with diabetes induced by STZ exhibited Depression-like behaviors frequently, and their wounds healed more slowly. The number of c-Fos LHb neurons in diabetic mice was significantly increased, and STZ-induced diabetic mice had increased neuronal firing of LHb neurons compared to healthy mice.

Our experiment is the first to phenotypically discover an improvement in depression-like behavior in diabetic mice, with positive effects on diabetic non-healing wounds. The underlying mechanisms of why improving depression can accelerate wound healing remain largely unexplored. Our research revealed that reducing LHb neural activity enhanced both wound healing and depression-like behavior in diabetic mice (Figure 5). In 2023, Li et al. (2023) found that the blockade of NK1R may be therapeutic not only for depression but also for diabetic non-healing wounds. So, whether the activity of LHb is in some way related to this receptor will guide our future research work. Our findings provide fresh insights for the clinical treatment of chronic wounds, which are not limited to traditional single-target treatments but use macro-control to accelerate the process of wound healing.

## 5 Conclusion

This study establishes a significant link between diabetes-induced depression-like behavior and the activity of the LHb region. We demonstrate that inhibiting neuronal activity in the LHb can effectively alleviate depression-like symptoms caused by diabetes. Importantly, this intervention not only addresses psychological distress but also notably accelerates the healing process of wounds in diabetic models.

## Data availability statement

The original contributions presented in the study are included in the article/Supplementary material, further inquiries can be directed to the corresponding author.

## Ethics statement

The animal study was approved by the Laboratory Animal Ethics Committee of Jinan University. The study was conducted in accordance with the local legislation and institutional requirements.

## Author contributions

ZD: Data curation, Methodology, Writing—original draft, Writing—review & editing. JW: Formal analysis, Methodology, Supervision, Validation, Writing—original draft, Writing—review & editing. HC: Software, Validation, Writing—review & editing. JL: Funding acquisition, Resources, Writing—review & editing.
